# Comparative effectiveness of 4 natural and chemical activators of Nrf2 on inflammation, oxidative stress, macrophage polarization, and bactericidal activity in an *in vitro* macrophage infection model

**DOI:** 10.1371/journal.pone.0234484

**Published:** 2020-06-08

**Authors:** Malika Ali, Marcel Bonay, Valentin Vanhee, Stéphane Vinit, Therese B. Deramaudt

**Affiliations:** 1 UVSQ, INSERM END-ICAP, Université Paris-Saclay, Versailles, France; 2 Service de Physiologie-Explorations Fonctionnelles, Hôpital Ambroise Paré, APHP, Boulogne, France; University of the Pacific, UNITED STATES

## Abstract

Inflammation plays a crucial role in the defense response of the innate immune system against pathogen infection. In this study, we selected 4 compounds for their potential or proven anti-inflammatory and/or anti-microbial properties to test on our *in vitro* model of bacteria-infected THP-1-derived macrophages. We first compared the capacity of sulforaphane (SFN), wogonin (WG), oltipraz (OTZ), and dimethyl fumarate (DMF) to induce the nuclear factor erythroid 2-related factor 2 (Nrf2), a key regulator of the antioxidant, anti-inflammatory response pathways. Next, we performed a comparative evaluation of the antioxidant and anti-inflammatory efficacies of the 4 selected compounds. THP-1-derived macrophages and LPS-stimulated macrophages were treated with each compound and expression levels of genes coding for inflammatory cytokines IL-1β, IL-6, and TNF-α were quantified by RT-qPCR. Moreover, expression levels of genes coding for M1 (IL-23, CCR7, IL-1β, IL-6, and TNF-α) and M2 (PPARγ, MRC1, CCL22, and IL-10) markers were determined in classically-activated M1 macrophages treated with each compound. Finally, the effects of each compound on the intracellular bacterial survival of gram-negative *E*. *coli* and gram-positive *S*. *aureus* in THP-1-derived macrophages and PBMC-derived macrophages were examined. Our data confirmed the anti-inflammatory and antioxidant effects of SFN, WG, and DMF on LPS-stimulated THP-1-derived macrophages. In addition, SFN or WG treatment of classically-activated THP-1-derived macrophages reduced expression levels of M1 marker genes, while SFN or DMF treatment upregulated the M2 marker gene MRC1. This decrease in expression of M1 marker genes may be correlated with the decrease in intracellular *S*. *aureus* load in SFN- or DMF-treated macrophages. Interestingly, an increase in intracellular survival of *E*. *coli* in SFN-treated THP-1-derived macrophages that was not observed in PBMC-derived macrophages. Conversely, OTZ exhibited pro-oxidant and proinflammatory properties, and affected intracellular survival of *E*. *coli* in THP-1-derived macrophages. Altogether, we provide new potential therapeutic alternatives in treating inflammation and bacterial infection.

## Introduction

Inflammation is a defense response of the innate immune system triggered by pathogen and non-pathogen infections or by tissue damage. This acute and coordinated inflammatory mechanism serves in the resolution of infection or tissue repair, followed by the return to homeostasis. Macrophages are important components of the innate immunity and play a major role in the maintenance of cell homeostasis and host cell defense system by modulating the inflammatory response and phagocytosis. Depending on the surrounding environment, macrophages can adopt very distinct functional phenotypes, including a classically activated phenotype (M1) and an alternatively activated phenotype (M2). M1 macrophages are characterized by a production of proinflammatory cytokines, chemokines, and reactive oxygen and nitrogen species (ROS/RNS) [1]. Conversely, M2 macrophages are characterized by a production of anti-inflammatory cytokines, chemokines, and activation of antioxidant and anti-inflammatory signaling pathways, thus favoring tissue healing and a return to homeostasis [2,3].

Dysregulation in the coordinated inflammatory process may be detrimental to the host, and subsequently lead to chronic inflammatory diseases [4]. The anti-inflammatory drugs currently used in the treatment of acute or chronic inflammatory disorders are of the non-steroidal type and carry a variety of systemic adverse effects [5]. Therefore, finding natural or synthetic compounds with a different anti-inflammatory mechanism of action may be of great interest. Nuclear factor erythroid 2-related factor (Nrf2) plays a central role in the regulation of the antioxidant and anti-inflammatory responses. Under homeostatic conditions, Nrf2 is sequestered in the cytoplasm by Kelch-like ECH-associated protein 1 (Keap1), and led to ubiquitin-dependent proteasomal degradation [6,7]. Under cellular stress, Nrf2 is released from Keap1 and translocates into the nucleus, where it heterodimerizes with small Maf proteins and binds antioxidant response elements (ARE) located in the upstream regulatory regions of target genes coding for anti-inflammatory, antioxidant, and cytoprotective proteins [7,8]. Nrf2-mediated anti-inflammatory response is thought to be ROS-dependent, although a recent report showed a direct inhibitory effect of Nrf2 on the recruitment of RNA polymerase II, preventing the transcription of genes coding for the proinflammatory cytokines IL-1β, IL-6 [9,10]. Furthermore, activation of Nrf2 signaling pathway in phagocytic cells improved their anti-viral and anti-bacterial functions [11–13]. Based on the literature, we selected 4 pharmacological compounds, found to modulate Nrf2 signaling, with anti-inflammatory properties: sulforaphane (SFN), wogonin (WG), oltipraz (OTZ), and dimethyl fumarate (DMF) [14–17].

SFN, a well-established activator of Nrf2, is a natural isothiocyanate found in cruciferous vegetables with antioxidant, anti-inflammatory, and chemoprotective effects [18]. The molecular basis for SFN activated anti-inflammatory mechanism is suggested to occur via activation of the Nrf2 signaling pathway and inhibition of NF-κB [19]. SFN treatment of LPS-stimulated microglial cells increased expression of Nrf2/HO-1 and reduced NF-κB signaling, leading to a decrease in expression levels of the proinflammatory genes TNF-α, IL-1β, IL-6, concomitant with an increase in expression levels of IL-10 and IL-4 [20]. Moreover, SFN-mediated activation of Nrf2 triggers the synthesis of phase II cytoprotective enzymes [21].

WG (5,7-dihydroxy-8-methoxyflavone) is a natural flavonoid extracted from the roots of *Scutellaria baicalensis*. In a mouse model of colorectal carcinogenesis, WG treatment of dextran sulfate sodium-induced mice reduced inflammation by promoting nuclear translocation of Nrf2 and inhibiting NF-κB nuclear translocation and phosphorylation [22]. Furthermore, WG was found to suppress inflammation in IL-1β-stimulated human osteoarthritis chondrocytes and cartilage explants by inducing a moderate oxidative stress and by blocking Keap-1 interaction with Nrf2, leading to activation of Nrf2 signaling pathway [23].

OTZ (5-(2-pyrazinyl)-4-methyl-1,2-dithiol-3-thione), a synthetic dithiolethione initially used for treating *Schistosoma Mansoni* infection, has been reported to possess chemoprotective properties [24]. In mouse Hepa1c1c7 hepatoma cell line, OTZ activated Nrf2 signaling pathway by promoting Nrf2 translocation into the nucleus and by inducing transcriptional expression of ARE-dependent genes coding for phase II enzymes [25]. Oral administration of OTZ to a mouse model of urinary bladder carcinoma reduced cancer incidence by increasing phase II detoxifying enzymes activity in an Nrf2-dependent manner [26].

DMF is a fumaric acid ester approved by the Food and Drug Administration (FDA) for the treatment of patients with moderate-to-severe psoriasis and by the European Medicines Agency (EMA) for the treatment of patients with relapsing-remitting multiple sclerosis (RRMS) [27,28]. For instance, in a rat model of autoimmune neuritis, treatment with DMF showed a decrease in proinflammatory M1 macrophages and an increase in anti-inflammatory M2 macrophages. DMF elevated Nrf2 and HO-1 expression levels, while proinflammatory genes IFN-γ, TNF-α, IL-6, IL-17 were downregulated and anti-inflammatory genes IL-4 and IL-10 were upregulated [29]. DMF has also been found to increase Nrf2 nuclear translocation and expression of Nrf2 target genes, and decrease NF-κB-mediated inflammation in a mouse model of sickle cell disease [30].

In this study, we performed a head-to-head comparison of 2 natural compounds, SFN and WG, and 2 synthetic compounds, DMF and OTZ, on THP-1-derived macrophages and primary PBMC-derived macrophages. We first tested the cytotoxicity of the 4 selected compounds used in our *in vitro* LPS-induced inflammation and confirmed that each compound activated Nrf2. Next, we investigated the capacity of the selected compounds to modulate the proinflammatory activities of classically-induced M1 macrophages. Finally, we evaluated the bactericidal activity of THP-1-derived macrophages and PBMC-derived macrophages treated with each selected compound upon infection with either gram-negative *E*. *coli* or gram-positive *S*. *aureus*.

## Material and methods

### Reagents and compounds

Phorbol 12-myristate 13-acetate (PMA), human interferon gamma (IFNγ), lipopolysaccharides (LPS), dimethyl sulfoxide (DMSO), sulforaphane (SFN), dimethyl fumarate (DMF), Oltipraz (OTZ) were purchased from Sigma (MERCK). Wogonin (WG) and anti-GAPDH antibody were obtained from Millipore (MERCK). Dual Luciferase (Firefly-Renilla) Assay System was purchased from BPS Bioscience. RPMI (Roswell Park Memorial Institute) 1640 culture medium, phosphate-buffered saline (PBS), fetal bovine serum, and human GM-CSF were obtained from Eurobio Scientific. Tryptic soy broth and tryptic soy agar were from Conda laboratories (Dutscher). Lipofectamine^®^ LTX & PLUS^™^ Reagent, Live/Dead^™^ BacLight^™^ bacterial viability kit, CellROX^™^ Green Oxidative Stress Reagent, Maxima First Strand cDNA synthesis kit, and Trizol were obtained from ThermoFisher scientific. Fluoromount-G^™^ (EMS) was purchased from Euromedex. DC Protein Assay Reagents and iTaq SYBR green supermix were purchased from Bio-Rad. Anti-Lamin A/C antibody was purchased from Abcam. Anti-Nrf2 antibody was obtained from ProteinTech. IRDye^®^ 680CW goat anti-mouse IgG, and IRDye^®^ 800CW Goat anti-Rabbit IgG secondary antibodies were purchased from *LI-COR*^®^ Biosciences.

### Cell culture and cell differentiation

Human monocytic THP-1 cell line (ATCC^®^ TIB-202^™^) was maintained in RPMI 1640 medium supplemented with 10% heat-inactivated fetal bovine serum, 10 mM HEPES buffer, 1 mM sodium pyruvate, and 50 μM of β-mercaptoethanol in a humidified incubator containing 5% CO_2_ at 37 °C. THP-1 were differentiated into macrophages by incubating the cells in medium supplemented with 10 μM PMA for 48 h. PMA stimulation was reduced to 24 h when followed by LPS/IFNγ stimulation.

Human peripheral blood mononuclear cells (PBMCs) were Ficoll-isolated from heparinized blood of healthy donors purchased from Etablissement Français du Sang (Paris, France). Isolated monocytes were differentiated into macrophages by addition of 20 ng/ml GM-CSF to the RPMI 1640 medium supplemented with 10% of heat-inactivated fetal bovine serum, 10 mM HEPES buffer, and 1 mM sodium pyruvate, and incubated for 7 days prior to treatment.

### LPS stimulation and LPS/IFNγ-mediated M1 polarization of macrophages

For LPS stimulation, THP-1-derived macrophages cultured in 6-well plates at 1 × 10^6^ cells/well were pretreated with vehicle (DMSO), 10 μM SFN, 25 μM WG, 25 μM OTZ, or 20 μM DMF. After 3 h, LPS 5 ng/ml was added to the medium. After 4 h of additional incubation, total RNAs were extracted for analysis.

For M1 polarization of THP-1-derived macrophages, 24 hours THP-1-derived macrophages were rinsed twice with sterile PBS, then fresh culture medium supplemented with LPS 100 ng/ml and 20 ng/ml IFNγ were added for 48 h to obtain proinflammatory macrophages (M1). After medium change, unpolarized and M1 polarized THP-1-derived macrophages were treated with DMSO, 10 μM SFN, 25 μM WG, 25 μM OTZ, or 20 μM DMF for an additional 48 h.

### Nrf2 activity assay

Activation of Nrf2 was quantified using ARE-Reporter kit (BPS Bioscience). Briefly, 7.5 × 10^4^ THP-1 cells per well were seeded in 96-well plates and differentiated with PMA. After 48 h incubation, THP-1-derived macrophages were transfected with ARE-Firefly luciferase reporter vector mixed with constitutively-expressed Renilla luciferase reporter vector using the Lipofectamine^®^ LTX & PLUS^™^ Reagent (Thermo Fisher) following the manufacturer’s instructions, and incubated for 24 h. Transfected THP-1-derived macrophages were treated with SFN, WG, OTZ, or DMF for an additional 48 h. Chemiluminescence signals from the dual luciferase Firefly-Renilla assay system were measured on the FLUOstar Omega microplate reader (BMG Labtech). Nrf2 activity was obtained from the ratio of firefly luminescence signal to the corresponding Renilla luminescence signal.

### Quantification of intracellular ROS production

ROS production was measured using the CellROX^™^ Green Oxidative Stress kit (Thermo Fisher). Briefly, 2.5 × 10^5^ cells/well of THP-1 were seeded on coverslips placed in 24-well plates and cells were differentiated with PMA for 48 h. Macrophages were treated with vehicle (DMSO) or selected compound (SFN, WG, OTZ, DMF) for 7 h. For LPS stimulation, macrophages were treated with DMSO or selected compound for 3 h prior to addition to the medium of 5 ng/ml LPS and an additional 4 h incubation. Macrophages were stained with CellROX reagent for 30 min, washed with PBS and fixed with 4% paraformaldehyde (PFA). Nuclei were counterstained with 4′, 6-diamidino-2-phenylindole (DAPI), and cells were mounted with the Fluoromount-G aqueous mounting medium. Fluorescent images were taken using Leica SP8 confocal microscope (Leica Microsystems) and fluorescent signals were analyzed from selected 7 fields per treatment using Image J 1.48v (National Institutes of Health).

### Bacteria strains and growth culture

*Escherichia coli* strain (ATCC 25922) and *Staphylococcus aureus* strain (ATCC 25923) were grown aerobically in Trypticase soy broth to the optical density of 1 at 37 °C under agitation. Bacterial glycerol stocks were prepared and when required, frozen stocks were thawed and bacteria were diluted in sterile PBS at the appropriate concentration. For intracellular bacterial survival assay, bacteria were seeded on Trypticase soy broth solidified with 1.5% agar.

### Intracellular bacterial survival assay

THP-1-derived macrophages and PBMC-derived macrophages, seeded in 24-well plates at 2.5 × 10^5^ cells/well and 5 × 10^5^ cells/well, respectively, were treated with DMSO or chemical compound for 48 h prior to infection with the gram-negative bacteria *E*. *coli*, or the gram-positive bacteria *S*. *aureus* at an MOI of 10. Gentamycin was added to the cell culture medium 1 h after infection to inhibit extracellular bacteria growth, and cells were incubated for an additional 24 h before colony forming unit (CFU) assay. Briefly, CFU assay consists in washing cells twice with PBS and lyzing cells in 1 ml ice-cold sterile water. Numeration of intracellular bacteria was obtained by plating 5-fold serial dilutions on Trypticase soy agar plates and incubating at 37 °C for 24 h.

### Bacteria viability

Viability of *S*. *aureus* was assessed using the Live/Dead^™^ BacLight^™^ Bacterial Viability kit (Thermo Fisher) according to the manufacturer’s recommendations. SYTO9 was used to stain bacteria and Propidium Iodide to stain membrane-damaged bacteria. Stained bacteria were incubated with each compound for up to 90 min. Fluorescent signals were monitored on the FLUOstar Omega microplate reader (BMG Labtech) at 45, 60, and 90 min using 488 nm excitation and measuring emission signals at 530 nm and 630 nm.

### Subcellular fractionation and immunoblotting

THP-1-derived macrophages, at 1 × 10^6^ cells/well and 3 × 10^6^ cells for total protein extraction and nuclear fractionation respectively, were treated with DMSO or selected compound (SFN, WG, OTZ, DMF) for 6 h.

For total protein extraction, treated cells were washed twice with cold PBS and lyzed with RIPA (150 mM NaCl, 1% Triton X-100, 0.5% sodium deoxycholate, 0.1% SDS, 50 mM Tris-HCl, pH 7.5) supplemented with a cocktail of protease inhibitors. After 30 min on ice, the cell lysates were centrifuged at 12,000 x g for 5 min at 4 °C and the supernatants containing the protein extracts were isolated.

For subcellular fractionation, treated cells were washed twice with cold PBS and lyzed with buffer A (10 mM HEPES, 1.5 mM MgCl_2_, 10 mM KCl, 0.5 mM DTT, 0.05% NP40, pH 7.9) supplemented with a cocktail of protease inhibitors. After 10 min incubation on ice, the cell lysates were centrifuged at 900 x g for 15 min at 4 °C. The supernatants containing the cytoplasmic fractions were discarded and the cell pellets containing the nuclear fractions were resuspended in buffer B (5 mM HEPES, 1.5 mM MgCl_2_, 0.2 mM EDTA, 0.5 mM DTT, 26% glycerol, pH 7.9) supplemented with a cocktail of protease inhibitors. After 30 min on ice and 5 passages through a 29 G syringe, the mixtures were centrifuged at 10,000 x g for 10 min at 4 °C. The supernatants containing the nuclear fractions were then collected.

Protein concentrations were quantified using the DC protein assay kit (Bio-Rad) and 20 μg of protein were resolved by SDS-PAGE on 4–20% gradient gels. After protein transfer on polyvinylidene difluoride membranes (Immobilon-FL, Merck), western blots were performed using the iBind Flex western system (Invitrogen, Thermo Fisher) following the manufacturer’s instructions. Briefly, antibodies against Nrf2, GAPDH, lamin A/C, IRDye680RD, and IRDye800RD were diluted in iBind Flex FD solution. Fluorescence signals were acquired using Odyssey CLx imaging system (LI-COR) and densitometric analysis was done using de Image Studio Lite software 4.0v. Immunoblots shown are representative of at least three independent experiments.

### RNA extraction and real-time quantitative PCR analysis

Total RNA was extracted using Trizol reagent following the manufacturer’s protocol. The concentration of total RNA was determined using GE NanoVue spectrophotometer (GE Healthcare). One μg of total RNA was reverse transcribed into cDNA using the Maxima First strand cDNA synthesis kit following the manufacturer’s instructions. cDNA was analyzed by real-time quantitative PCR (RT-qPCR) using the CFX384 thermocycler and iTaq SYBR green supermix. Each sample was done in triplicate. The specific primers synthetized by Eurogentec are listed in Table 1. CCR7 and MRC1 genes code for C-C chemokine receptor type 7 CD197 and mannose receptor CD206, respectively. Gene expression levels were normalized to the corresponding expressions of the reference gene 18S ribosomal RNA. Data were analyzed on Bio-Rad CFX manager 3.1 using the ΔΔCt method [31].

**Table 1:** List of primers used for qPCR analysis.

Target genes	Forward primer sequences (5’-3’)	Reverse primer sequences (5’-3’)
IL-23	GTTCCCATACCAGTGTGG	GAGGCTTGGAATCTGCTGAG
CCR7	GATTACATCGGAGACAACACCA	AGTACATGATAGGGAGGAACCAG
IL-1β	AATGATGGCTTATTACAGTGGCA	GTCGGAGATTCGTAGCTGGA
IL-6	GTAGCCGCCCCACACAGA	CATGTCTCCTTTCTCAGGGCTG
TNF-α	GGAGAAGGGTGACCGACTC	TGGGAAGGTTGGATGTTCGT
PPARγ	TTCAGAAATGCCTTGCAGTG	CCAACAGCTTCTCCTTCTCG
MRC1	ATGGGTGTCCGAATCTCAG	CGATCCCTTGTAGAGCATA
CCL22	ATTACGTCCGTTACCGTCTG	TAGGCTCTTCATTGGCTCAG
IL-10	TCAAGGCGCATGTGAACTCC	GATGTCAAACTCACTCATGGCT
18S rRNA	GATAGCTCTTTCTCGATTCCG	CTAGTTAGCATGCCAGAGTC

### Statistical analysis

Data are expressed as mean ± standard errors of the mean (SEM). All experiments were performed independently from at least 3 independent experiments. All statistical comparisons were tested using Student's unpaired *t*-test and considered significant at a value of p < 0.05.

## Results

### The selected 4 compounds increased Nrf2 nuclear translocation and activity

**Fig 1:**
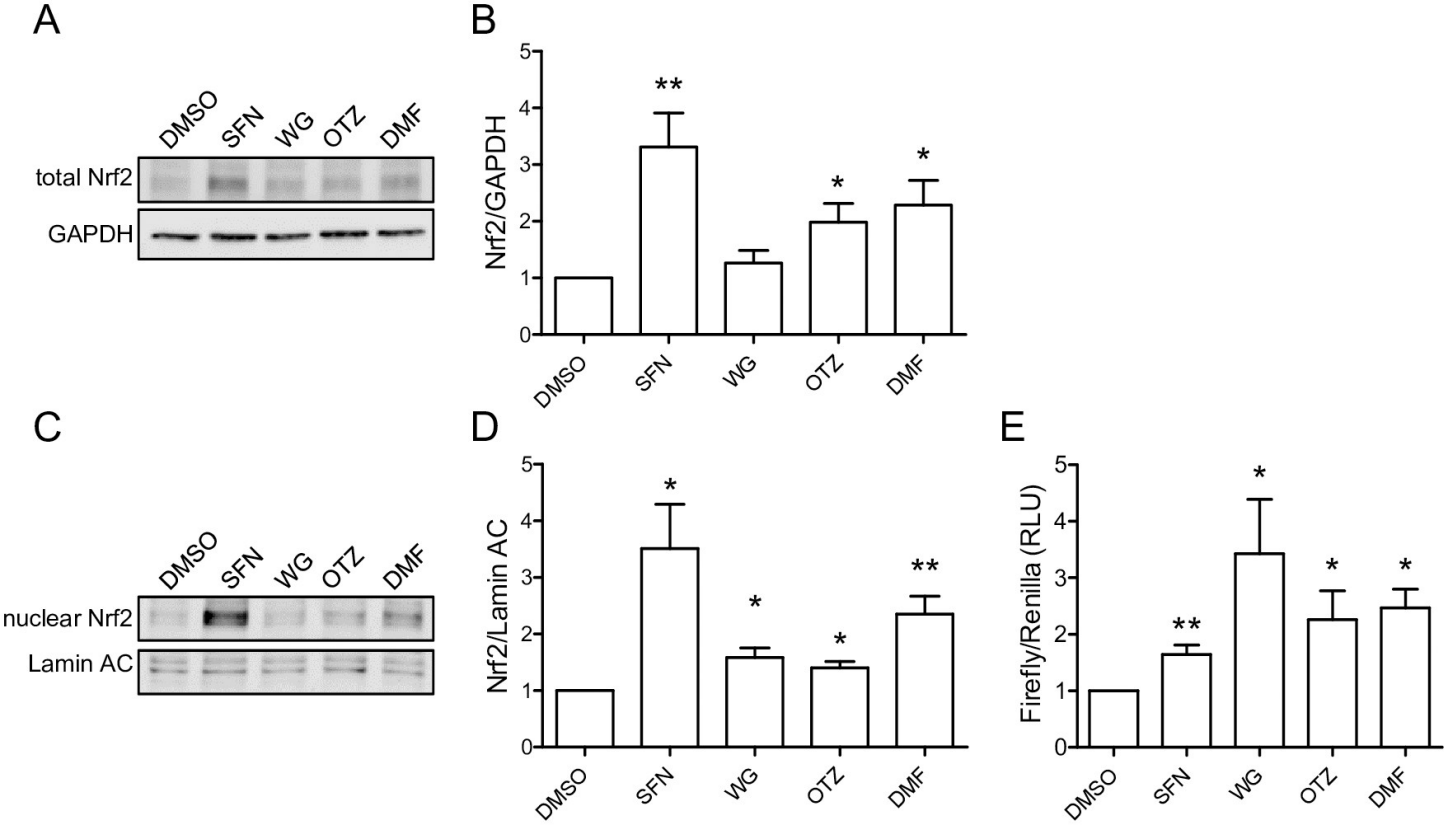
SFN, WG, OTZ, and DMF activate Nrf2. THP-1-derived macrophages were treated with 10 μM SFN, 25 μM WG, 25 μM OTZ, 20 μM DMF or DMSO for 6 h. (A, B) Proteins from whole cell lysates were examined by Western blot analysis and Nrf2 levels were detected using specific Nrf2 and GAPDH antibodies. Images are representative of 5 independent experiments. Densitometric analysis was performed using Image Studio Lite 4.0v, and Nrf2 signals were normalized to the corresponding GAPDH values. (C, D) Immunoblots of nuclear Nrf2 and Lamin A/C proteins are representative of 5 independent experiments. Graph shows nuclear Nrf2 normalized to the corresponding Lamin A/C values. (E) THP-1-derived macrophages, transfected with ARE-Luciferase vector for 24 h, were treated with SFN, WG, OTZ, DMF or DMSO for 24 h. Expression of ARE-Luciferase reporter was assessed by chemiluminescent assay (n = 3 independent experiments done in triplicate). Data are presented as mean ± SEM. *p< 0.05, **p< 0.01.

In order to compare the effect of the multifunctional compounds SFN, WG, OTZ, and DMF on Nrf2, THP-1-derived macrophages were incubated with specific concentrations of SFN (10 μM), WG (25 μM), OTZ (25 μM), and DMF (20 μM) selected after dose-dependent response assays (S1 Fig) according to those used in the literature [23,32–35]. We first validated the non-toxic concentration of each compound by MTT assay. After 24 h treatment, none of the compounds affected macrophages viability compared to vehicle DMSO-treated macrophages (S2 Fig). To investigate the activating effect of SFN, WG, OTZ, and DMF on Nrf2, THP-1-derived macrophages were treated for 6 h with either natural or synthetic compound. Western blot analysis of total protein lysates revealed a significant increase in Nrf2 protein level in THP-1-derived macrophages treated with SFN, OTZ, and DMF, while treatment with WG did not modulate Nrf2 protein level when compared to DMSO treated macrophages (Fig 1A and 1B). Translocation of Nrf2 in the nucleus was then evaluated by Western blot analysis and showed that although WG, OTZ, or DMF significantly increased nuclear Nrf2, treatment with SFN was most potent in inducing Nrf2 translocation (Fig 1C and 1D). Next, Nrf2 activity was evaluated in THP-1-derived macrophages transfected with an ARE-luciferase reporter vector and treated with each compound. The chemiluminescent signals were captured with a spectrophotometer after 24 h. A significant increase in Nrf2/ARE-dependent transcription was detected for each compound-treated macrophages compared to DMSO treated macrophages (Fig 1E). These data confirm that the activation of Nrf2 by each compound is heterogeneous.

### SFN, WG, OTZ and DMF modulate ROS production

**Fig 2:**
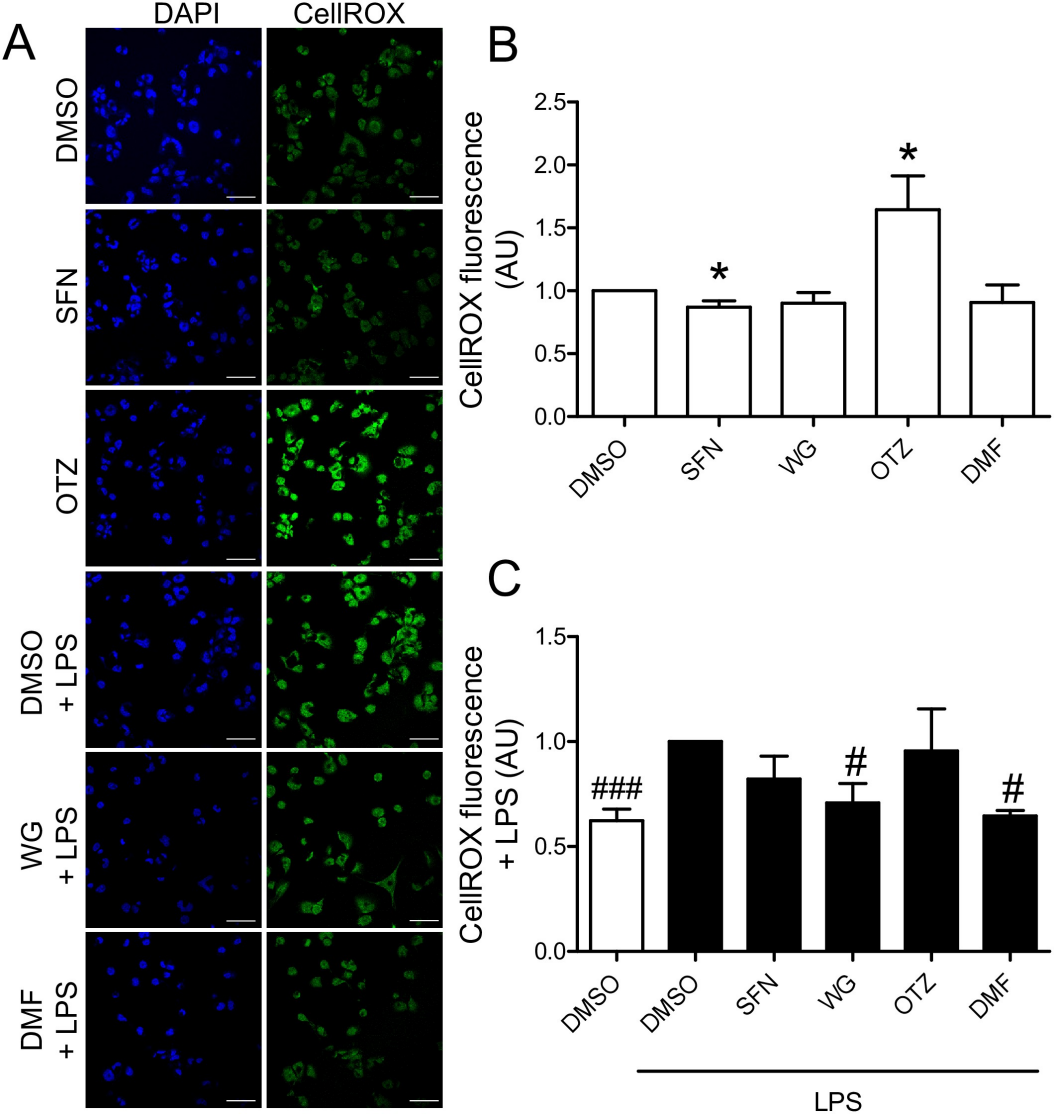
Effects of the selected compounds on ROS production. THP-1-derived macrophages were treated with SFN, WG, OTZ, DMF or DMSO for 3 h prior to addition of low-dose LPS (5 ng/ml) to the cell culture medium. After an additional 4 h incubation, live cells were stained with CellROX reagent. After PFA fixation, nuclei were counterstained with DAPI. For each condition, seven images of intracellular ROS labeled macrophages were taken by confocal microscopy and CellROX fluorescent signals were analyzed using Image J software (n = 3 independent experiments). Scale bar = 50 μm. *p< 0.05 compared to DMSO, ^#^p< 0.05 compared to DMSO+LPS.

Since Nrf2 is a well-known regulator of antioxidants, we sought to determine the cellular oxidative stress in THP-1-derived macrophages treated with SFN, WG, OTZ, or DMF and CellROX Green reagent was added in order to measure oxidative stress. After 6 h of treatment, cells were PFA-fixed, counterstained with DAPI, and images of CellROX-stained macrophages were acquired by confocal microscopy (Fig 2A). Analysis of fluorescent signals showed a significant decrease in ROS levels in THP-1-derived macrophages treated with SFN (Fig 2B). Conversely, treatment of macrophages with OTZ promoted ROS production. LPS stimulation was used on macrophages to examine the effect of each compound on LPS-induced oxidative stress. LPS was added to the culture medium of THP-1-derived macrophages 3 h after pretreatment with DMSO or the indicated compound. After an additional 4 h incubation, ROS levels were determined (Fig 2C). We found that LPS-mediated increase of intracellular ROS levels were significantly reduced by pretreating THP-1-derived macrophages with WG or DMF compared to that seen in DMSO-pretreated macrophages. Interestingly, the decrease in ROS levels were not as significant in SFN pretreated macrophages. These data suggest that treatments with WG or DMF efficiently repress LPS-induced oxidative stress.

### SFN, WG, and DMF reduce LPS-induced inflammation

**Fig 3:**
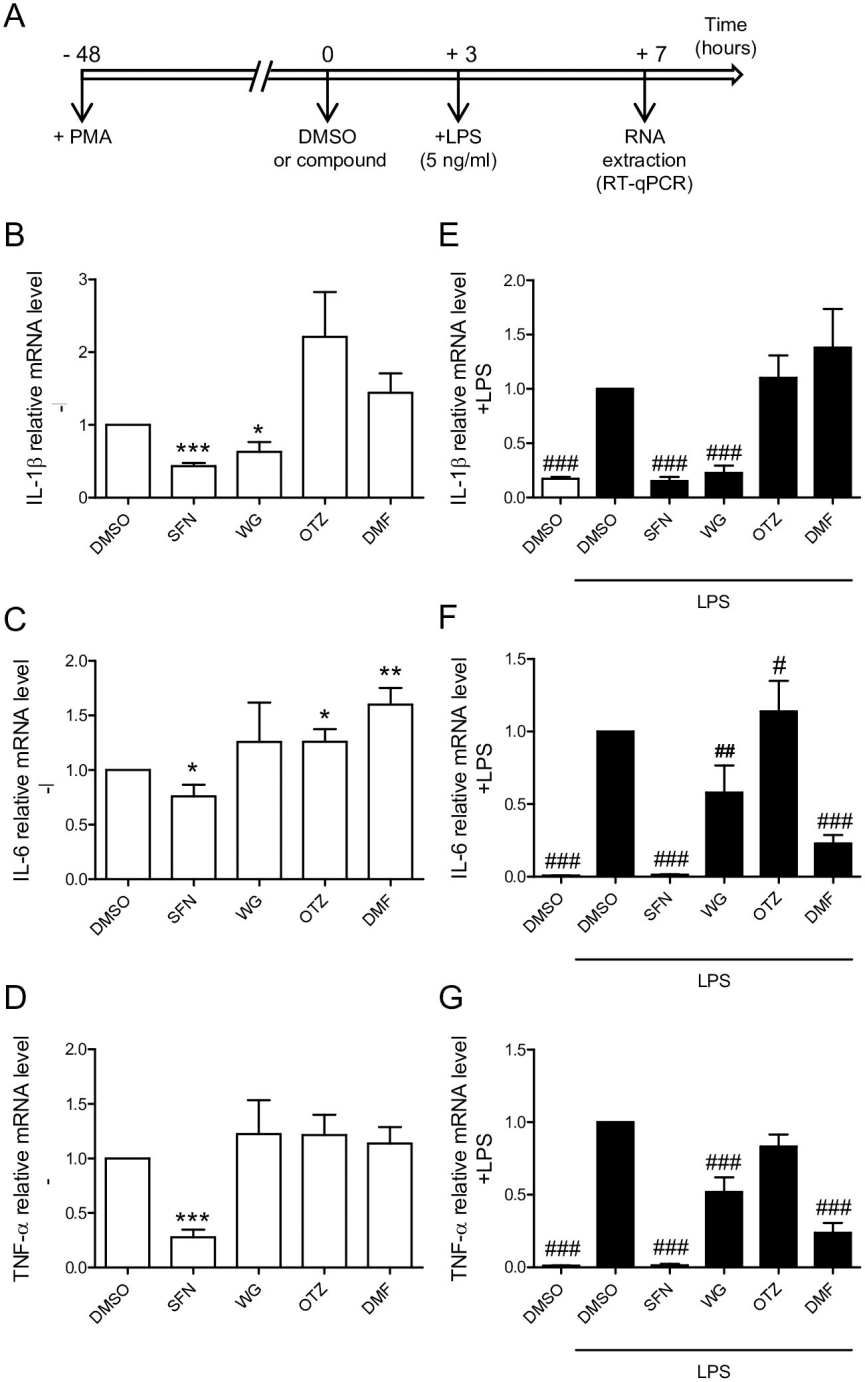
Effects of the selected compounds on genes coding for the proinflammatory cytokines IL-1β, IL-6, and TNF-α. (A) Experimental design. THP-1-derived macrophages, treated with SFN, WG, OTZ, DMF or DMSO for 3 h, were stimulated or not with low-dose LPS and incubated for an additional 4 h. Cells were lyzed and total RNA was extracted. Expression levels of IL-1β (B, E), IL-6 (C, F) and TNF-α (D, G) genes were quantified by RT-qPCR. Fold changes were normalized to DMSO treated THP-1-derived macrophages (n = 6 independent experiments done in triplicate). *p< 0.05, **p< 0.01, ***p< 0.001 compared to DMSO, and ^#^p< 0.05, ^##^p< 0.01, ^###^p< 0.001 compared to DMSO+LPS.

To determine the effect of each compound on inflammation, THP-1-derived macrophages were pretreated with DMSO, SFN, WG, OTZ, or DMF prior to addition or not of LPS (Fig 3A). Total RNAs were extracted from the macrophages incubated for 7 h with each indicated compound then the expression levels of the genes coding for the proinflammatory cytokines IL-1β, IL-6, and TNF-α were analyzed by RT-qPCR. In THP-1 cells differentiated into macrophages with PMA, SFN significantly repressed IL-1β, IL-6, and TNF-α gene expressions WG treatment affected IL-1β gene expression (Fig 3B–3D). Notably, transcriptional expression level of IL-6 was increased by treating the macrophages with OTZ or DMF (Fig 3C). In the context of LPS-induced inflammation, LPS-mediated increase of IL-1β, IL-6, and TNF-α gene expressions was repressed by pretreating with SFN or WG as compared to LPS-stimulated macrophages treated with DMSO. DMF decreased IL-6 and TNF-α mRNA expression levels but did not repress IL-1β gene expression (Fig 3E–3G). OTZ did not impact on LPS-induced expression levels of IL-1β, IL-6, and TNF-α.

### M1/M2 marker profile in THP-1-derived macrophages treated with SFN, WG, OTZ, and DMF

**Fig 4:**
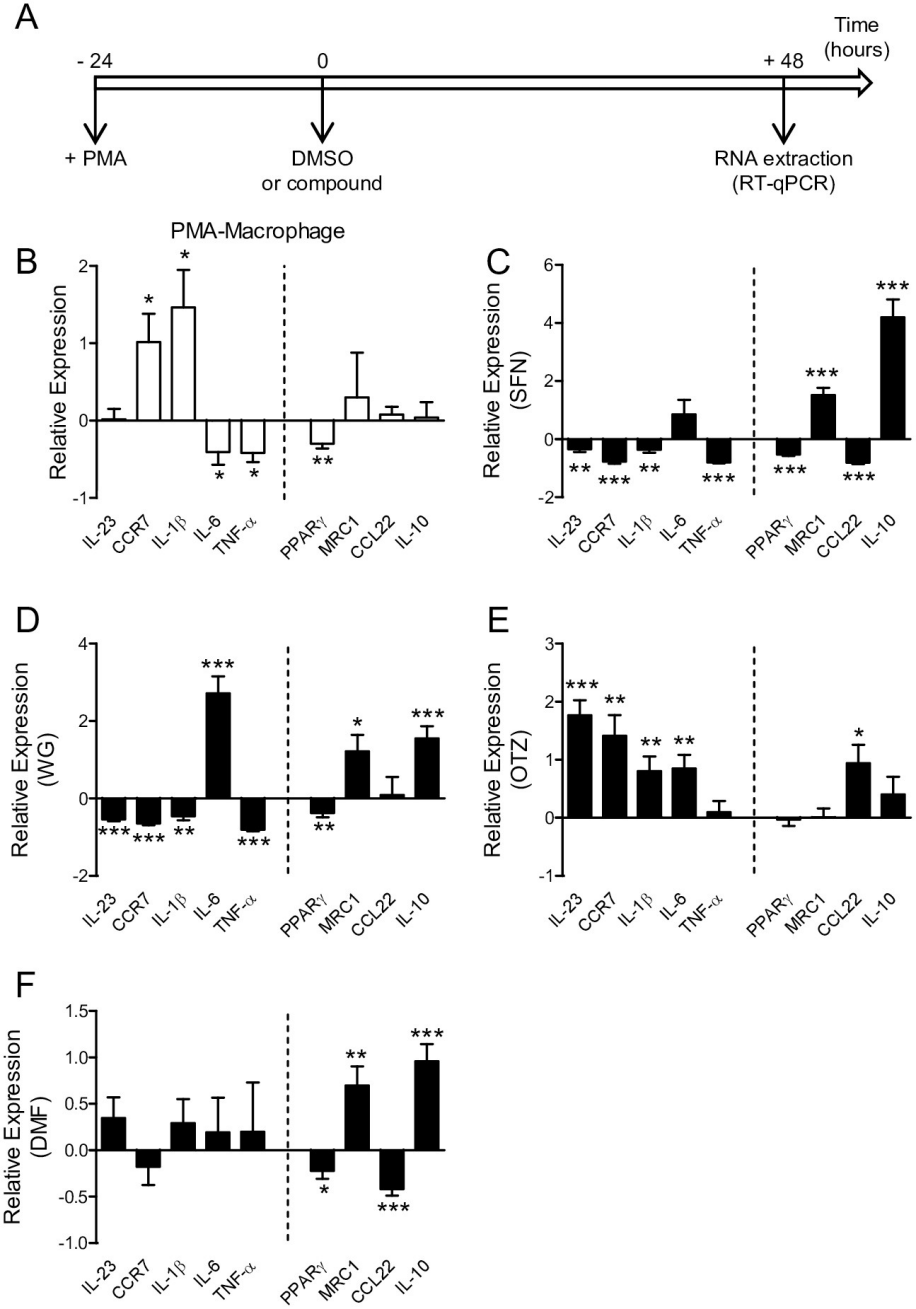
Effects of the 4 selected compounds on THP-1-derived macrophages. (A) Experimental design. THP-1-derived macrophages were differentiated for 24 h with PMA, then the culture medium was replaced with fresh medium containing DMSO, SFN, WG, OTZ or DMF. Total RNA was extracted 48 h after treatment and gene expression levels were determined by RT-qPCR. Each graph shows on the left side of the dash line M1 marker genes (IL-23, CCR7, IL-1β, IL-6, and TNF-α), and on the right side M2 marker genes (PPARγ, MRC1, CCL22, and IL-10). (B) Fold-change mRNA expression levels in DMSO-treated macrophages were normalized to untreated THP-1-derived macrophages. (C-F) Fold-change mRNA expression levels in SFN (C), WG (D), OTZ (E), and DMF (F) -treated macrophages were normalized to DMSO-treated macrophages (at least n = 4 experiments done in triplicate). *p< 0.05, **p< 0.01, ***p< 0.001.

Since macrophages may be classically or alternatively activated by their surrounding environment, we explored the possibility that SFN, WG, OTZ, and DMF treatment could elicit M1/M2 phenotypic changes by examining the expression levels of M1 (IL-1β, IL-6, TNF-α, IL-23, CCR7) or M2 (IL-10, PPARγ, MRC1, CCL22) marker genes. PMA-differentiated THP-1-derived macrophages were incubated in presence of DMSO or the selected compound for 48 h (Fig 4A). After RNA extraction, RT-qPCR analysis demonstrated that THP-1-derived macrophages treated with DMSO presented a specific gene signature in M1/M2 marker genes compared to untreated THP-1-derived macrophages, including the upregulation of two M1 marker genes (CCR7 and IL-1β), and the downregulation of two M1 marker genes (IL-6 and TNF-α), and one M2 marker gene PPARγ (Fig 4B). Treatment with SFN or WG resulted in the negative regulation of 4 M1 marker genes (IL-23, CCR7, IL-1β, and TNF-α) and 2 M2 marker genes (PPARγ and CCL22), and the positive regulation of the M2 marker genes MRC1 and IL-10 (Fig 4C and 4D). In agreement with the pro-oxidative effect of OTZ and its lack of anti-inflammatory properties (Fig 2), OTZ significantly increased all M1 marker genes but TNF-α gene (Fig 4E). DMF treatment did not modulate M1 marker genes, while MRC1 and IL-10 gene expressions were upregulated as compared to DMSO treated macrophages (Fig 4F). These findings suggest that treatments with SFN and WG inhibit M1 marker genes and proinflammatory gene expressions. Similar to SFN, treatment with WG and DMF upregulate gene expression of M2 markers (MRC1 and IL-10).

### M1/M2 gene signature in M1 polarized THP-1-derived macrophages treated with Nrf2 activators

**Fig 5:**
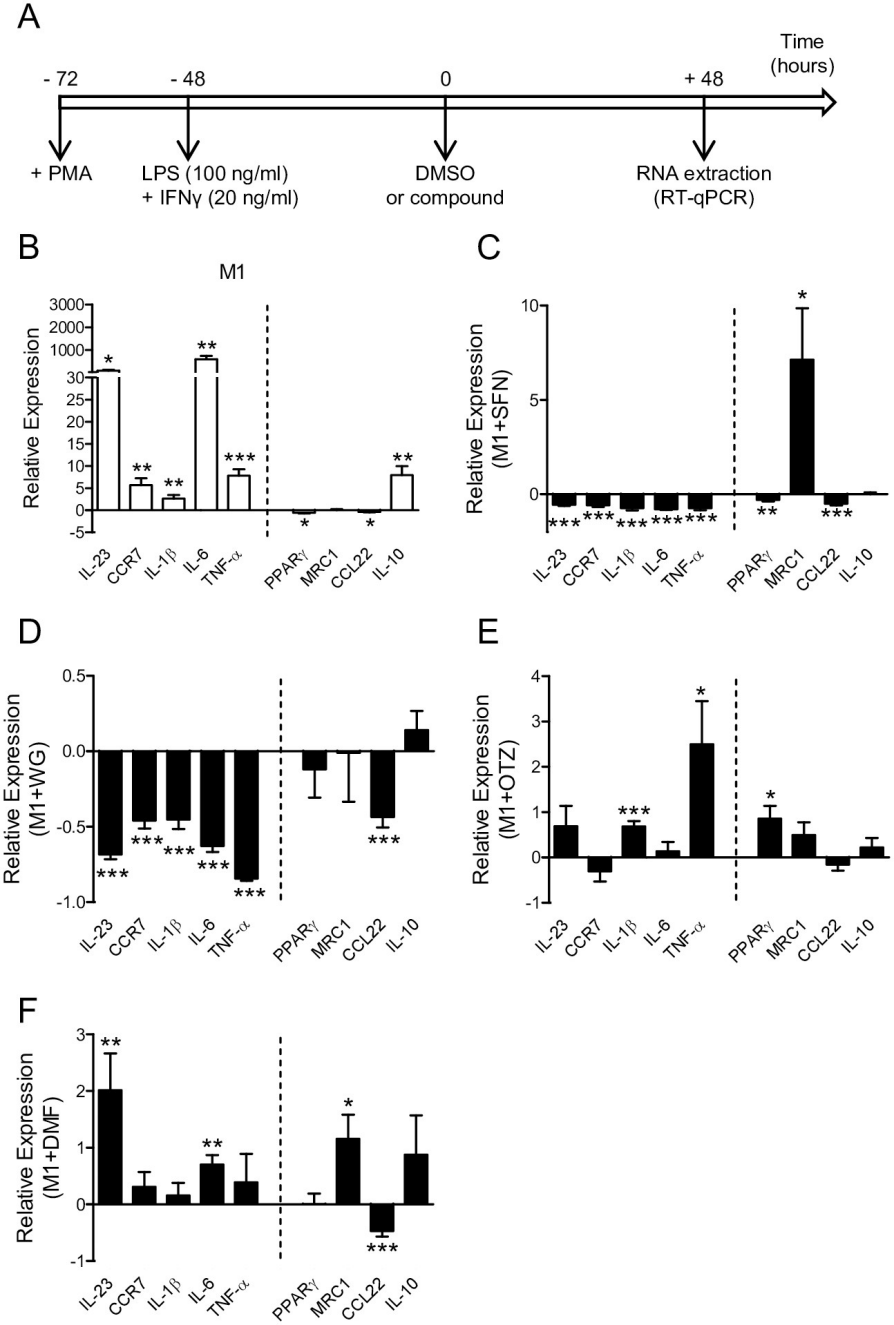
Effects of the 4 selected compounds on classically activated THP-1-derived macrophages. (A) Experimental design. THP-1-derived macrophages were differentiated for 24 h with PMA, then stimulated with high-dose LPS and IFNγ. After 48 h, the culture medium was replaced with fresh medium containing DMSO, SFN, WG, OTZ or DMF. Total RNA was extracted 48 h after treatment and gene expression levels were determined by RT-qPCR. Each graph shows on the left side of the dash line the relative expression of M1 marker genes (IL-23, CCR7, IL-1β, IL-6, and TNF-α), and on the right side that of M2 marker genes (PPARγ, MRC1, CCL22, and IL-10). (B) Fold-change mRNA expression levels in DMSO-treated LPS/IFNγ-stimulated (M1) macrophages were normalized to DMSO-treated unstimulated THP-1-derived macrophages. (C-F) Fold-change mRNA expression levels in M1 polarized macrophages treated with SFN (C), WG (D), OTZ (E) or DMF (F) were normalized to M1-polarized macrophages treated with DMSO (at least n = 4 experiments done in triplicate). *p< 0.05, **p< 0.01, ***p< 0.001.

Next, we examined whether treatment with SFN, WG, OTZ or DMF may impact on the polarization of macrophages to M1 mediated by LPS/IFNγ treatment. THP-1-derived macrophages were activated with high dose of LPS and IFNγ prior to medium change and treatment with DMSO or the selected compound (Fig 5A). After 48 h treatment, RNAs were extracted and analyzed by RT-qPCR. Data showed that stimulation with LPS/IFNγ classically activated THP-1 derived macrophages leading to the upregulation of M1 marker genes IL-23, CCR7, IL-1β, IL-6, TNFα, and of M2 marker gene IL-10 compared to non-activated macrophages (Fig 5B). Treatment of M1 macrophages with SFN or WG resulted in a decreased expression of the genes coding for M1 markers (Fig 5C, 5D and 5G), while treatment with OTZ and DMF resulted in a robust M1 marker profile compared to DMSO treated M1 macrophages (Fig 5E and 5F). Interestingly, the modulatory effect of each compound on M2 marker genes was heterogeneous. In addition, SFN and DMF significantly increased expression of M2 marker gene MRC1, which codes for mannose receptor CD206 in M1 macrophages. Altogether, these data suggest that SFN and WG are able to significantly reduce the expression level of M1 marker genes in M1 polarized macrophages, while SFN and DMF are able to upregulate the expression level of several M2 marker genes.

### SFN, OTZ, and DMF regulate intracellular survival of *E*. *coli* and *S*. *aureus*

**Fig 6:**
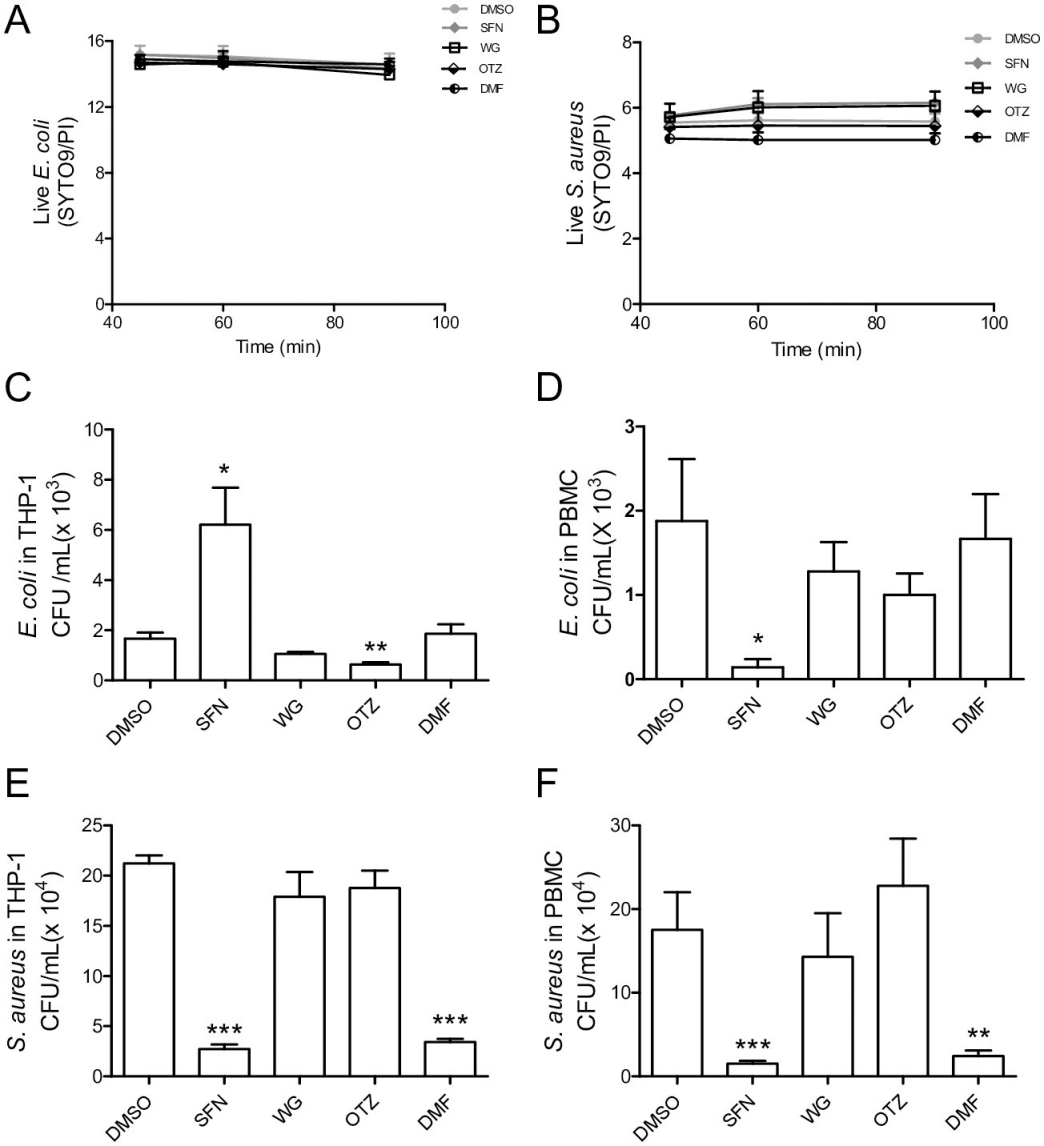
SFN, OTZ, and DMF affect the bactericidal activity of macrophages. Bacterial viability was assessed using the BacLight bacterial viability kit. Bacteria were stained with SYTO9 and dead bacteria with propidium iodide (PI). (A) *E*. *coli* and (B) *S*. *aureus* were incubated for 90 minutes in PBS complemented with DMSO, SFN, WG, OTZ or DMF and fluorescent signals were measured at 45, 60, and 90 minutes by spectrophotometry (n = 3 independent experiments done in triplicate). (C, D) THP-1-derived macrophages (C) and human PBMC-derived macrophages (D) were pretreated with DMSO, SFN, WG, OTZ or DMF for 24 h prior to 1 h infection with *E*. *coli* at MOI 10, then gentamycin was added to the medium for the remaining incubation time. Intracellular survival of *E*. *coli* was determined 24 h after infection by means of CFU counts (n = 5 independent experiments). (E, F) THP-1-derived macrophages (E) and PBMC-derived macrophages (F) were submitted to the same protocol of treatment and infection with *S*. *aureus* at MOI 10. CFU counts of *S*. *aureus* was determined 24 h after infection (n = 3 and 5 independent experiments, respectively). *p< 0.05, **p< 0.01, ***p< 0.001.

To evaluate the effect of each Nrf2 activator on macrophage activity, intracellular bacterial survival was examined. THP-1-derived macrophages pretreated with each selected compound were infected with either the gram-negative bacterium *E*. *coli* or the gram-positive bacterium *S*. *aureus*. We first assessed the toxicity of each compound on the bacterial viability using the Live/Dead bacteria viability kit. Spectrophotometer analysis showed that the viability of *E*. *coli* or *S*. *aureus* was not significantly affected by the 4 compounds as compared to DMSO-treated bacteria after 90 minutes incubation (Fig 6A and 6B). The survival of intracellular bacteria was determined by colony forming unit (CFU) assay 24 hours after infection with *E*. *coli* of THP-1-derived macrophages pretreated with DMSO or the selected compound. Bacterial counts showed that SFN promoted *E*. *coli* survival compared to DMSO-treated macrophages infected with *E*. *coli*, while treatment with OTZ effectively reduced intracellular *E*. *coli* survival (Fig 6C). WG and DMF had no effect on intracellular *E*. *coli* persistence. Noticeably, infection with *E*. *coli* of PBMC-derived macrophages pretreated with SFN revealed a significant decrease in bacterial intracellular survival (Fig 6D). WT, OTZ, and DMF did not significantly modified *E*. *coli* survival in PBMC-derived macrophages. Interestingly, the effects of SFN, WG, OTZ, and DMF differed in macrophages infected with *S*. *aureus*. Data showed that SFN and DMF significantly decreased intracellular *S*. *aureus* survival, while pretreatment with WG and OTZ showed no effect on intracellular *S*. *aureus* load (Fig 6E). These effects observed in THP-1-derived macrophages infected with *S*. *aureus* were also validated in primary macrophages derived from human peripheral blood monocyte cells (Fig 6F). WG had no effect on the bactericidal activity of infected macrophages. Taken together, these data suggest that SFN has opposite effect on the bactericidal activity of THP-1-derived macrophages and primary PBMC-derived macrophages in the context of *E*. *coli* infection, while the effect of DMF appears bacteria-dependent.

## Discussion

Macrophages are innate immune cells that play an important role as the first line of defense against invading pathogens or tissue damage. In the present study, we used LPS stimulation of THP-1-derived macrophages as a model of inflammation to compare 4 selected activators of Nrf2, known for their anti-inflammatory properties, and we investigated the multiple effects of each compound on macrophage oxidative stress, inflammation, and bactericidal activity. We first confirmed that each compound was able to activate Nrf2, by verifying its translocation in the nucleus, and positively regulate the expression level of an ARE-dependent reporter gene. We also demonstrated that SFN, WG, and DMF decreased, with relative effectiveness, the oxidative stress and the expression of genes coding for the proinflammatory cytokines IL-1β, IL-6, and TNF-α in LPS-stimulated THP-1-derived macrophages. Current literature supports the notion that M1 macrophages are functionally polarized in response to bacterial components and host mediators [36]. However, a prolonged M1 status may result in enhanced inflammation, tissue injury, and organ failure, which calls for a rapid resolution of the inflammatory response and a restoration of cell homeostasis. Our data show that pretreatment of classically activated M1 macrophages with SFN or WG successfully interfered with LPS/IFNγ-mediated M1 polarization of THP-1-derived macrophages as shown by the inhibition of the transcriptional expression of M1 marker genes. Unlike the other 3 compounds, we found that OTZ treatment increases oxidative stress in macrophages and exhibits no anti-inflammatory property.

Among the 4 tested compounds, SFN was the most potent anti-inflammatory compound. The anti-inflammatory activity of SFN has been extensively studied and multiple molecular targets have already been identified. One possible molecular mechanism that may partially participate in the anti-inflammatory activity of SFN, is the targeting of NF-κB by SFN leading to a reduction in the DNA-binding effectiveness of NF-κB [19]. Another suggested mechanism for the suppression of LPS-mediated inflammation by SFN may be through the covalent modification of the cysteine residues located in the extracellular domain of Toll-like receptor 4 (TLR4) by SFN, which prevents TLR4 oligomerization and thereby LPS-mediated activation of downstream TLR signaling, consequently decreasing the activation of downstream NF-κB and mitogen-activated protein kinases (MAPK) signaling pathways [37–39]. Furthermore, SFN-mediated activation of Nrf2 signaling pathway probably induces expression of ARE-dependent genes participating in the anti-inflammatory responses, such as the Nrf2/HO-1 axis [40]. Activated Nrf2 is also thought to bind in the vicinity of genes coding for inflammatory cytokines including IL-6 and IL-1β, and interfere with their transcription [9]. Of note, our *in vitro* model of LPS stimulation shows that the repressive effect of SFN on LPS-induced oxidative stress was not as pronounced as that of WG or DMF.

Our comparative studies underlined the strong suppressive effects of SFN on inflammation and M1 polarization. The M1/M2 gene signature observed in M1 polarized macrophages after SFN treatment leads to the switch in polarization from M1-like phenotype triggered by LPS/IFNγ stimulation to an M2-like phenotype. DMF treatment increased expression of the M2 marker gene MRC1 without modifying the LPS/IFNγ-induced M1/M2 marker profiles. Our results were not consistent with the reduction in inflammation in a rodent model of neurodegenerative diseases (Guillain-Barré syndrome) treated with DMF, where a switch from spleen M1-macrophages toward M2 phenotype was observed [29], although additional analysis of other M1/M2 marker genes may be required to ascertain our findings. Interestingly, despite the anti-inflammatory property of WG, treatment with WG had no effect on the bactericidal activity of THP-1-derived macrophages infected with either *E*. *coli* or *S*. *aureus*.

Macrophages are essential in the process of eradicating microbial invasion, but paradoxically are frequently targeted by intracellular pathogens to evade detection and be used as reservoir for dissemination. We find here that the effect of SFN, OTZ, and DMF on the bactericidal activity of THP-1-derived macrophages may be bacteria-dependent. We hypothesize that the ability of *E*. *coli* or *S*. *aureus* to persist and proliferate within the macrophage plays a major role in their intracellular survival. The laboratory strain *E*. *coli* ATCC 25922 is a gram-negative bacterium that harbors no resistance against internalization, phagolysosome maturation, and intracellular killing in macrophages. It is then reasonable to conclude that switch from M1 phenotype to M2 phenotype by SFN treatment may provide a more hospitable niche for *E*. *coli* intracellular survival. Conversely, since M1 polarization of macrophages is important in the process of eradicating internalized bacteria, the proinflammatory environment and high level of oxidative stress generated by OTZ treatment could participate in the decrease of *E*. *coli* burden in THP-1-derived macrophages. Additional experiments will be needed to investigate the opposite effect of THP-1-derived macrophages and PBMC-derived macrophages on *E*. *coli* intracellular survival when treated with SFN. *S*. *aureus* strain ATCC 25923, a gram-positive opportunistic bacterium that has been previously shown to persist within macrophages [41]. Based on the existing literature, intracellular pathogens might be able to tolerate enhanced inflammation in macrophages, or evolve strategies to interfere with phagosome maturation, cell apoptosis, or interfere with M1 polarization [36,42]. Therefore, we speculated that reversion of the M1-like phenotype triggered by SFN treatment of THP-1-derived macrophages and PMBC-derived macrophages may activate a mechanism involved in the decrease of intracellular *S*. *aureus* intracellular. Additional experiments investigating cellular and molecular functions, such as phagocytosis or phagosomal maturation, will be necessary to identify the molecular mechanisms activated by DMF or derivatives [43].

In conclusion, this comparative study establishes the strong antioxidant, anti-inflammatory, and bactericidal effects of SFN, and to a lesser extent DMF, on macrophages. We hypothesize that the survival of extracellular bacteria such as *E*. *coli* and intracellular bacteria such as *S*. *aureus* relies on the oxidative and inflammatory environment within the macrophage. Thus, modulation of the antioxidant and anti-inflammatory responses in macrophages using selected Nrf2 activators may provide new therapeutic alternatives for treating inflammation and bacterial infection.

## Supporting information

S1 Fig Dose-dependent response assays. Western blot analysis was done after 24 h treatment of THP-1-derived macrophages with the DMSO vehicle, SFN (10 μM), WG (10, 25, 50, 75 μM), OTZ (1, 5, 10, 25 μM), and DMF (1, 5, 10, 20, 40 μM). HO-1 and GAPDH signals were quantified by densitometric analyses using Image J 1.4v. Immunoblots are representative of 3 to 5 independent experiments. *p< 0.05, **p< 0.01.
(PDF)

S2 Fig Cell viability assay. The cytotoxicity of each compound was assessed by MTT assay. THP-1-derived macrophages were incubated with each compound for 24 h (n = 3 independent experiments done in triplicates).
(PDF)

S1 Raw images (PDF)

S1 Data (PDF)
