# Supplementary figures and images for: Comparative effectiveness of 4 natural and chemical activators of Nrf2 on inflammation, oxidative stress, macrophage polarization, and bactericidal activity in an in vitro macrophage infection model
Source: PLoS One. 2020 Jun 8;15(6):e0234484. doi: 10.1371/journal.pone.0234484 (PMC7279588; doi:10.1371/journal.pone.0234484)

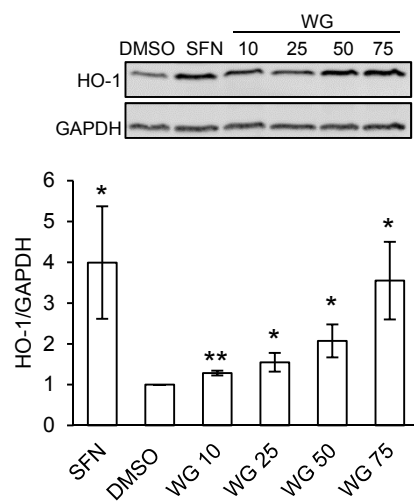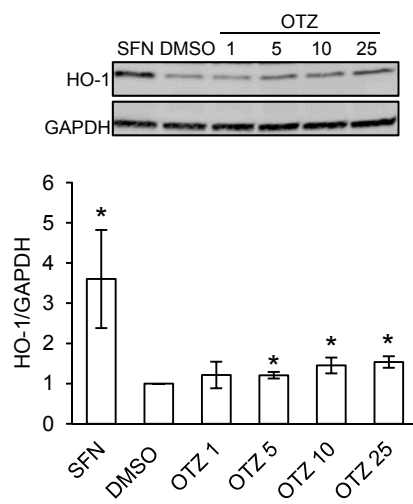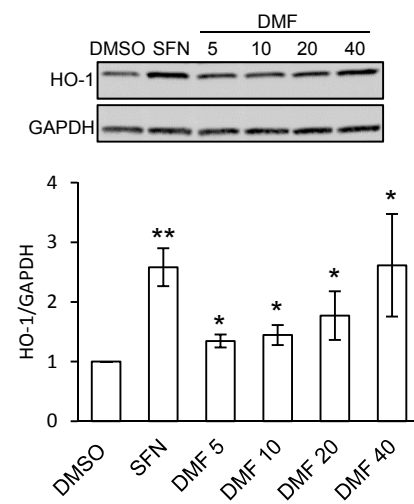

Supplement: S1 Fig — Western blot analysis was done after 24 h treatment of THP-1-derived macrophages with the DMSO vehicle, SFN (10 μM), WG (10, 25, 50, 75 μM), OTZ (1, 5, 10, 25 μM), and DMF (1, 5, 10, 20, 40 μM). HO-1 and GAPDH signals were quantified by densitometric analyses using Image J 1.4v. Immunoblots are representative of 3 to 5 independent experiments. *p< 0.05, **p< 0.01. (PDF) [file pone.0234484.s001.pdf]

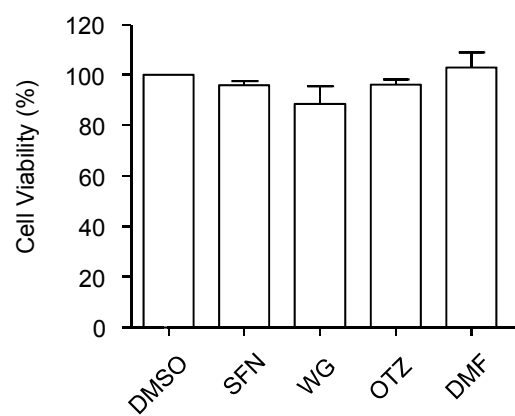

Supplement: S2 Fig — The cytotoxicity of each compound was assessed by MTT assay. THP-1-derived macrophages were incubated with each compound for 24 h (n = 3 independent experiments done in triplicates). (PDF) [file pone.0234484.s002.pdf]
