# Supplementary material for: Comparative effectiveness of 4 natural and chemical activators of Nrf2 on inflammation, oxidative stress, macrophage polarization, and bactericidal activity in an in vitro macrophage infection model
Source: PLoS One. 2020 Jun 8;15(6):e0234484. doi: 10.1371/journal.pone.0234484 (PMC7279588; doi:10.1371/journal.pone.0234484)
Supplement: S1 Raw images — (PDF) [file pone.0234484.s003.pdf]

total protein lysates

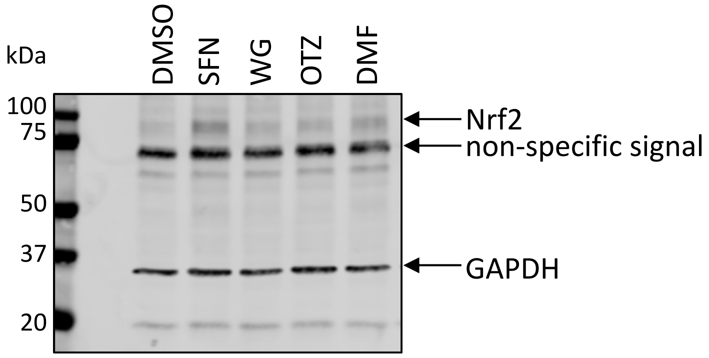

nuclear protein lysates

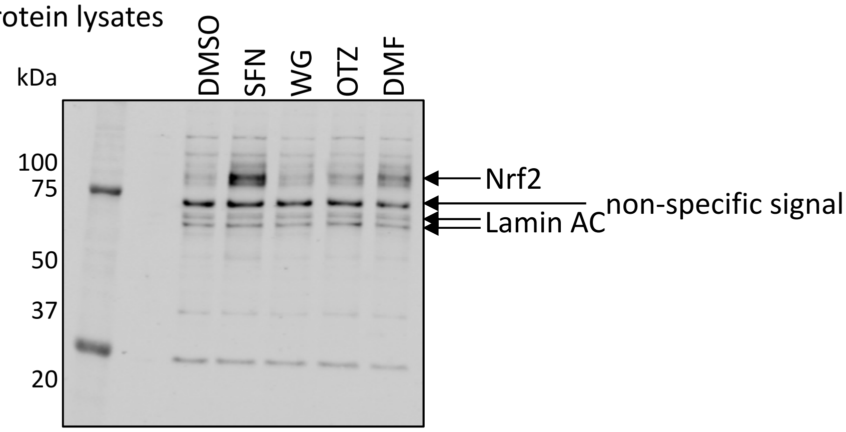

Fig 1 raw: images acquired using Odyssey scanner (Li-Cor) and Image Studio software.

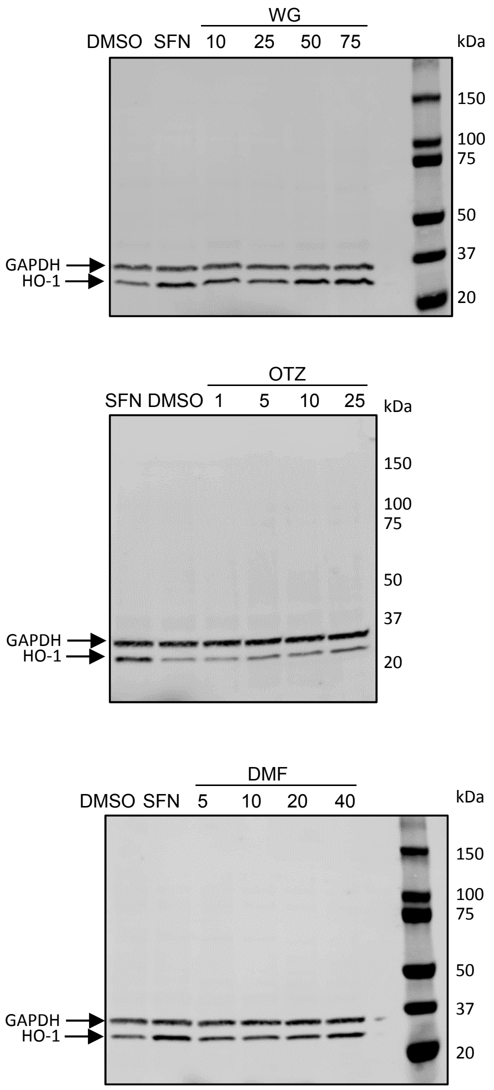

Fig S1 raw: images acquired using Odyssey scanner (Li-Cor) and Image Studio software.
