# Supplementary material for: Comparative effectiveness of 4 natural and chemical activators of Nrf2 on inflammation, oxidative stress, macrophage polarization, and bactericidal activity in an in vitro macrophage infection model
Source: PLoS One. 2020 Jun 8;15(6):e0234484. doi: 10.1371/journal.pone.0234484 (PMC7279588; doi:10.1371/journal.pone.0234484)
Supplement: S1 Data — (PDF) [file pone.0234484.s004.pdf]

Data for Fig 1: total and nuclear Nrf2

Fig 1B	**exp1**	**exp2**	**exp3**	**exp4**	**exp5**	**mean**	**SEM**
DMSO	1	1	1	1	1	1	0
SFN	2.26041852	3.4902312	3.40470895	2.00869516	5.39365817	3.3115424	0.66979886
WG	0.83836663	0.98181646	1.19144165	1.19144165	2.1112373	1.26286074	0.24863695
OTZ	1.42692817	2.61384472	2.46156473	0.93602292	2.46156473	1.97998506	0.37594422
DMF	1.37643288	1.61060407	3.35367482	1.72363281	3.35367482	2.28360388	0.49241492

Fig 1D	**exp1**	**exp2**	**exp3**	**exp4**	**exp5**	**mean**	**SEM**
DMSO	1	1	1	1	1	1	0
SFN	2.28956879	6.20103911	4.30188515	1.92235596	2.8307123	3.50911226	0.87821792
WG	1.93859865	1.60292019	1.39942958	1.0348861	1.9426457	1.58369605	0.19208019
OTZ	1.72075606	1.19217363	1.59423478	1.12381321	1.37153245	1.40050203	0.12769389
DMF	2.23919147	3.29064987	2.8366524	1.62792095	1.77067569	2.35301807	0.35274819

Data for Fig 1: ARE assays

Fig 1E	**exp1**	**exp2**	**exp3**
DMSO	1	1	1
SFN	1.513777	1.846154	2.076389
WG	5.076923	3.466667	1.742152
OTZ	1.650794	3.699605	1.6775
DMF	2.25	3.115385	2.046296

Data for Fig 2: CellRox signals

Fig 2B	**exp1**	**exp2**	**exp3**
DMSO	1	1	1
SFN	0.8952229	0.7331366	0.8818872
WG	0.6735459	0.970016	0.7450645
OTZ	1.188643	2.383627	1.69559
DMF	0.8826943	1.088296	0.9852031

Fig 2C	**exp1**	**exp2**	**exp3**	**exp4**
DMSO	0.4222941	0.5791487	0.6893133	0.7345473
DMSO+LPS	1	1	1	1
SFN+LPS	0.4456173	0.9462397	0.8441062	1.24494
WG+LPS	0.3933074	0.751395	0.7459654	0.9657744
OTZ+LPS	0.9430199	1.295971	1.111189	1.327773
DMF+LPS	0.7089916	0.6428923	0.6799901	0.6421083

Data for Fig 3: qPCR data sets

**Fig 3B**	**exp1**	**exp2**	**exp3**	**exp4**	**exp5**	**exp6**
DMSO	1	1	1	1	1	1
SFN	0.33551	0.34391	0.57467	0.3791	0.4177	0.54935
WG	0.89853	0.35135	1.1558	0.3907	0.43045	0.56615
OTZ	0.93842	0.81325	0.93269	2.979467	3.2826	4.317433
DMF	0.90687	0.79837	0.97303	1.682433	1.853617	2.43795

**Fig 3C**	**exp1**	**exp2**	**exp3**	**exp4**	**exp5**	**exp6**
DMSO	1	1	1	1	1	1
SFN	0.56961	1.02896	1.14869	0.52045	0.65661	0.62684
WG	1.12884	0.88775	3.01617	1.08306	0.63471	0.79925
OTZ	1.12147	1.39275	1.00179	1.73867	1.01891	1.28306
DMF	1.46346	2.13398	1.40896	1.97424	1.15696	1.4569

**Fig 3D**	**exp1**	**exp2**	**exp3**	**exp4**	**exp5**	**exp6**
DMSO	1	1	1	1	1	1
SFN	0.27914	0.50086	0.25468	0.34477	0.12926	0.13557
WG	0.91838	2.46052	1.21196	0.11256	1.28989	1.35284
OTZ	0.67191	0.67676	1.64781	1.12329	1.54421	1.61957
DMF	0.83634	0.55435	1.26701	1.2433	1.42769	1.49737

**Fig 3E**	**exp1**	**exp2**	**exp3**	**exp4**	**exp5**	**exp6**
DMSO	0.1762664	0.1042079	0.2202129	0.2121926	0.161427	0.1706485
DMSO+LPS	1	1	1	1	1	1
SFN+LPS	0.2235587	0.03316	0.2956424	0.1206527	0.1011259	0.1405973
WG+LPS	0.2040866	0.2532711	0.539275	0.1223078	0.1025142	0.1425341
OTZ+LPS	0.6208544	0.8847221	2.068041	1.011501	0.8477985	1.178763
DMF+LPS	0.4448154	0.5487964	0.8837408	2.134169	1.788765	2.487073

**Fig 3F**	**exp1**	**exp2**	**exp3**	**exp4**	**exp5**	**exp6**
DMSO	0.0099379	0.00251749	0.00219072	0.01021868	0.01013099	0.010285
DMSO+LPS	1	1	1	1	1	1
SFN+LPS	0.00754992	0.02099771	0.02480302	0.01450419	0.00842696	0.01077292
WG+LPS	0.4018559	0.0132581	0.2102327	1.229614	0.7144073	0.9132975
OTZ+LPS	1.334658	0.2306949	1.150434	1.774678	1.031082	1.318125
DMF+LPS	0.2675411	0.02147873	0.446433	0.2754271	0.1600231	0.2045717

**Fig 3G**	**exp1**	**exp2**	**exp3**	**exp4**	**exp5**	**exp6**
DMSO	0.01307588	0.006893	0.02394177	0.00805925	0.01018849	0.00832044
DMSO+LPS	1	1	1	1	1	1
SFN+LPS	0.00171072	0.05927016	0.01726824	0.00741628	0.00189862	0.00162623
WG+LPS	0.7013636	0.3668028	0.4443167	0.1307936	0.7916251	0.6780324
OTZ+LPS	0.9944549	0.6832944	0.7767825	0.5453131	1.076617	0.922129
DMF+LPS	0.5151355	0.09834658	0.3649049	0.122628	0.1780183	0.1524737

Data for Fig 4: qPCR data sets

**Fig 4B**	**exp1**	**exp2**	**exp3**	**exp4**
IL-23	0.4037	-0.10304	-0.03024	-0.2063
CCR7	2.02441	0.27483	0.91592	0.85216
IL-1β	2.8247	0.58055	1.03252	1.42189
IL-6	0.0856	-0.63939	-0.53873	-0.52962
TNF-α	-0.1347	-0.67774	-0.51898	-0.34502
PPARγ	-0.11378	-0.38484	-0.353	-0.34004
MRC1	-0.12558	2.00978	-0.11437	-0.56859
CCL22	0.27522	0.22786	-0.13167	-0.05777
IL-10	0.34508	-0.20495	0.40683	-0.39181

**Fig 4C**	**exp1**	**exp2**	**exp3**	**exp4**	**exp5**	**exp6**	**exp7**	**exp8**	**exp9**	**exp10**	**exp11**
IL-23	-0.51602	-0.47522	0.23128	0.37064	-0.2729	-0.52465	-0.54396	-0.2729	-0.67318	-0.52465	-0.54396
CCR7	-0.97198	-0.83677	-0.94476	-0.97868	-0.92097	-0.7959	-0.94709	-0.50829	-0.80939	-0.4817	-0.28982
IL-1β	-0.65232	-0.07174	0.23128	-0.52806	-0.05559	-0.44574	-0.69013	-0.05559	-0.61156	-0.44574	-0.69013
IL-6	-0.12635	4.46903	-0.02453	1.89406	2.42789	-0.61074	-0.16402	2.42789	-0.12602	-0.61074	-0.16402
TNF-α	-0.869	-0.75303	-0.60442	-0.83824	-0.71103	-0.86376	-0.87966	-0.71103	-0.8495	-0.86376	-0.87966
PPARγ	-0.67142	-0.51557	-0.70483	-0.56462	-0.50829	-0.4817	-0.28982	-0.50829	-0.80939	-0.4817	-0.28982
MRC1	0.42572	0.78219	1.68691	2.20162	1.06312	1.49637	1.47306	1.54986	2.99132		
CCL22	-0.8812	-0.77308	-0.95229	-0.88589	-0.93597	-0.55729	-0.76482	-0.93597	-0.93773	-0.55729	-0.76482
IL-10	6.63058	6.63058	5.77634	4.82188	3.0131	3.43496	4.82188	0.37449	3.0131	3.43496	

**Fig 4D**	**exp1**	**exp2**	**exp3**	**exp4**	**exp5**	**exp6**	**exp7**
IL-23	-0.73967	-0.47834	-0.33632	-0.6318117	-0.6053633	-0.50508	-0.484165
CCR7	-0.60886	-0.55465	-0.52661	-0.54546	-0.79932	-0.76722	-0.7409
IL-1β	-0.87278	-0.00575	-0.33632	-0.85375	-0.22586	-0.46983	-0.47026
IL-6	2.91888	0.45027	4.016	2.6901	3.7044	2.4128	2.8443
TNF-α	-0.61977	-0.74214	-0.92606	-0.81081	-0.85903	-0.87682	-0.83459
PPARγ	-0.84263	-0.42421	-0.61264	-0.31131	-0.22359	0.03829	-0.28107
MRC1	0.84672	-0.70606	2.07408	2.98588	1.02698	1.19904	1.07995
CCL22	-0.8812	-0.83368	-0.89833	-0.89675	1.01151	1.43123	1.69284
IL-10	1.7873	1.94225	2.4404	2.40483	1.39064	0.48948	0.39714

**Fig 4E**	**exp1**	**exp2**	**exp3**	**exp4**	**exp5**	**exp6**	**exp7**
IL-23	1.72283	0.91265	1.03659	1.39902	2.70471	2.22478	2.36105
CCR7	2.66086	0.9566	0.55873	1.83307	0.70472	0.55691	2.6131
IL-1β	2.15187	0.91265	0.18448	1.05252	0.40569	0.40454	0.51297
IL-6	0.69752	1.39062	0.54716	1.1	0.27195	0.09652	1.8317
TNF-α	-0.32423	0.02478	0.95755	-0.35515	0.53703	0.18633	-0.33753
PPARγ	0.44272	0.06834	0.0498	-0.35008	0.1053333	-0.1308667	-0.3982033
MRC1	0.34948	0.48565	-0.53021	0.4465333	-0.26438	-0.2019367	-0.2282033
CCL22	2.47498	1.72888	0.6094	0.1712267	0.41561	0.62435	0.5679367
IL-10	-0.45624	-0.45624	-0.03508	0.5680333	1.80643	0.74854	0.64014

**Fig 4F**	**exp1**	**exp2**	**exp3**	**exp4**	**exp5**	**exp6**	**exp7**
IL-23	1.42162	-0.43185	0.08664	0.15973	0.00579	0.558915	0.62479
CCR7	-0.78087	-0.51905	0.0421	-0.16943	0.51644	0.38495	-0.7148
IL-1β	0.08441	0.76461	-0.66836	-0.33352	0.50959	0.30138	1.38593
IL-6	1.07346	1.72614	0.78423	-0.71742	-0.32646	-0.48014	-0.7097
TNF-α	-0.73482	-0.57227	-0.36715	0.14372	-0.14779	3.32028	-0.25532
PPARγ	-0.47495	-0.43439	-0.03847	-0.25488	0.14439	-0.14779	-0.34889
MRC1	0.54321	1.3901	-0.18504	0.5463067	0.3410533	1.078553	1.166387
CCL22	-0.29626	-0.56442	-0.56017	-0.05582833	-0.51985	-0.4790933	-0.4515333
IL-10	0.66459	0.66459	1.23836	0.646818	1.947432	0.836392	0.722546

Data for Fig 5: qPCR data sets

**Fig 5B**	**exp1**	**exp2**	**exp3**	**exp4**	**exp5**	**exp6**	**exp7**
IL-23	277.3163	157.5865	165.2699	17.13525	24.10127	29.92663	29.6226
CCR7	10.00106	10.41112	9.34763	1.613696	1.64284	1.69182	4.94816
IL-1β	1.8615	1.31106	1.42018	1.15494	1.20004	6.27616	5.27772
IL-6	550.2258	1175.471	1197.189	220.5883	386.71	437.4566	169.9937
TNF-α	5.25875	6.55903	7.47688	8.18754	5.05335	16.10368	6.11249
PPARγ	-0.66838	-0.76232	-0.779	-0.8038854	-0.81995	0.72149	-0.47826
MRC1	0.27798	-0.34311	-0.7518093	0.72149	-0.20286	0.89765	-0.54847
CCL22	-0.26069	-0.39858	-0.8092142	-0.81711	-0.21582	-0.06757	-0.03119
IL-10	9.33686	7.13756	3.097185	0.5785	8.08889	16.92829	10.50383

**Fig 5C**	**exp1**	**exp2**	**exp3**	**exp4**	**exp5**	**exp6**	**exp7**
IL-23	-0.7425	-0.78399	-0.57553	-0.39854	-0.27127	-0.52591	-0.50097
CCR7	-0.87803	-0.77233	-0.80413	-0.46481	-0.34056	-0.4087	-0.379
IL-1β	-0.35846	-0.6946	-0.22309	-0.95569	-0.96966	-0.96968	-0.96734
IL-6	-0.87605	-0.83476	-0.97185	-0.72069	-0.64393	-0.74168	-0.70903
TNF-α	-0.33863	-0.86157	-0.82048	-0.20894	-0.87212	-0.90471	-1
PPARγ	-0.21869	-0.36036	0.06967	-0.23924	-0.46542	-0.49462	-0.38449
MRC1	1.60732	2.60655	1.10438	9.10543	10.09813	4.0577	21.38044
CCL22	-0.72343	-0.7202	-0.45554	-0.77327	-0.29359	-0.21986	-0.31214
IL-10	-0.28155	-0.36331	-0.08894	0.36937	0.24249	0.033	0.13166

**Fig 5D**	**exp1**	**exp2**	**exp3**	**exp4**	**exp5**	**exp6**	**exp7**
IL-23	-0.72483	-0.82842	-0.6555	-0.62634	-0.54728	-0.70547	-0.68998
CCR7	-0.28935	-0.59495	-0.68655	-0.47594	-0.35428	-0.421	-0.39192
IL-1β	-0.47371	-0.3332	-0.7647	-0.22586	-0.46983	-0.47026	-0.42936
IL-6	-0.73009	-0.45888	-0.76016	-0.63099	-0.52956	-0.65872	-0.61557
TNF-α	-0.85608	-0.89073	-0.77365	-0.81081	-0.85903	-0.87682	-0.83459
PPARγ	0.91225	0.08167	-0.27132	-0.23244	-0.46064	-0.49011	-0.37899
MRC1	1.77766	-0.62197	-0.24147	-0.37206	-0.31038	-0.68572	0.39069
CCL22	-0.34064	-0.23922	-0.69079	-0.19342	-0.54065	-0.49271	-0.55271
IL-10	0.28996	-0.2068	-0.41751	0.52461	0.38334	0.15011	0.25994

**Fig 5E**	**exp1**	**exp2**	**exp3**	**exp4**	**exp5**	**exp6**	**exp7**
IL-23	-0.28794	-0.87828	1.10112	-0.20854	2.46885	1.43752	1.2068
CCR7	-0.76588	0.98134	-0.71578	-0.4765	-0.35497	-0.42162	-0.39257
IL-1β	0.9083	0.4576	1.06806	1.05252	0.40569	0.40454	0.51297
IL-6	-0.6819	0.62557	0.80518	0.53585	0.14439	-0.34889	-0.12566
TNF-α	-0.47537	0.34423	-0.07963	5.6353	3.94411	3.32028	4.80151
PPARγ	0.68935	0.10689	-0.24468	1.97308	1.08915	0.97503	1.40543
MRC1	0.19466	0.49035	0.09845	0.38771	0.52402	-0.30546	2.07334
CCL22	-0.49569	-0.1129	-0.61396	0.50437	-0.14325	-0.05384	-0.16575
IL-10	0.37057	-0.08119	0.27589	0.43504	0.7682	0.66512	-0.892362

**Fig 5F**	**exp1**	**exp2**	**exp3**	**exp4**	**exp5**	**exp6**	**exp7**
IL-23	0.02625	2.19786	-0.6	3.30401	4.21478	2.39258	2.57105
CCR7	0.07408	1.55681	-0.80028	0.18662	0.46211	0.31104	0.37688
IL-1β	0.89494	0.30136	-0.84474	0.7256742	-0.2684157	0.2858315	-0.01759551
IL-6	1.18288	1.28284	-0.00348	0.43504	0.7682	0.5855	0.66512
TNF-α	0.50347	-0.3378	-0.51099	0.14372	-0.14779	3.32028	-0.25532
PPARγ	0.98679	0.1296	-0.48265	-0.25488	0.14439	-0.14779	-0.34889
MRC1	1.53253	3.17493	0.28346	1.59956	-0.12618	0.30106	1.32329
CCL22	-0.88622	-0.12	-0.37216	-0.70967	-0.4765	-0.35497	-0.39257
IL-10	0.20285	2.00622	1.0011	-0.42621	-0.53432	4.4614	-0.5857

Data sets for Fig 6:

Fig 6A

Time (min)	45	60	90
	13.26696	13.8397	13.32545
	14.14219	13.62321	12.71632
	16.01404	15.9303	15.6733
DMSO	15.34114	15.45342	14.68338
	15.45558	15.64376	14.86455
	15.02717	14.63365	13.58177
	17.60542	17.65081	17.31431
SFN	15.01757	14.59717	14.5412
	13.83637	14.23522	13.88092
	14.79607	14.34913	13.22337
	16.07348	16.06294	15.47717
WG	13.56626	13.83185	13.19507
	13.87724	13.84926	13.56734
	14.23217	14.35945	13.22307
	15.95822	15.65222	15.11304
OTZ	14.70924	14.4897	14.173
	14.87822	14.71364	14.21621
	14.58779	14.20053	13.26887
	15.36791	15.55261	15.29083
DMF	14.7822	14.63663	14.24383

Fig 6B

Temps (mins)	45	60	90
	5.540226	5.933168	5.958822
	6.093212	6.454601	6.365536
	5.546174	5.676034	5.838038
	5.116673	4.996951	4.803144
	5.682147	5.428635	5.537208
DMSO	5.30515	5.19819	4.988132
	5.531607	5.924263	6.015728
	5.923363	6.482346	6.352511
SFN	5.837804	5.926393	6.102956
	6.339965	6.516421	6.489696
	5.85297	6.493091	6.498774
WG	4.960276	5.044082	5.202806
	4.160935	4.246687	4.268859
	6.251901	6.678639	6.726513
	5.134539	5.202989	5.339323
	5.065913	4.931369	4.74429
OTZ	5.188464	5.025484	4.960412
	4.260376	4.378032	4.407699
	3.139159	4.210383	4.289407
	4.97052	5.022202	5.154311
	5.421749	5.19287	5.296728
DMF	5.578421	5.476883	5.211855

**Fig 6C**	**exp1**	**exp2**	**exp3**	**exp4**
DMSO	2.186667	0.9866667	1.74	1.726667
SFN	9.15	4.946667	8.18	4.54
WG	0.8933333	1.246667	1.066667	1.04
OTZ	0.4866667	0.8	0.5066667	0.76
DMF	2.62	1.6	0.9466667	2.286667

**Fig 6D**	**exp1**	**exp2**	**exp3**	**exp4**	**exp5**
DMSO	4.5	1.3	0.5	2.4	0.7
SFN	0.2	0.5	0	0	0
WG	0.9	1.8	1.6	0.1	2
OTZ	1.9	0.4	0.8	1.5	1.1
DMF	3.8	0	1.3	2.1	0.8

**Fig 6E**	**exp1**	**exp2**	**exp3**	**exp4**	**exp5**
DMSO	22.3	21.3	18.3	23	21.2
SFN	2.52	3.9	1.15	3.32	2.72
WG	9	23.8	18.3	20.5	17.9
OTZ	15.1	16.5	18.3	25.2	18.8
DMF	3.62	2.77	2.8	4.52	3.43

**Fig 6F**	**exp1**	**exp2**	**exp3**
DMSO	24.25	25.875	7.25
SFN	2.383333	1.6	1.28
WG	20	19	3.916667
OTZ	32.83333	22.25	13.25
DMF	3.85	3.35	1.283333

Data for Fig S1

Dose response after DMF treatment

HO-1	exp1	exp2	exp3	exp4
DMSO	1	1	1	1
SFN	2.11230313	2.12301741	3.20075477	2.88900821
DMF 5	1.24557261	1.5307438	1.4724721	1.13182916
DMF 10	1.06086793	1.39828781	1.73719713	1.58503499
DMF 20	1.14478582	2.33636364	2.42313238	1.17894953
DMF 40	1.72470893	2.29704272	4.8028705	1.62913712

Dose response after OTZ treatment

HO-1	exp1	exp2	exp3
SFN	2.11230313	5.49317677	3.20075477
DMSO	1	1	1
OTZ 1	1.03797717	1.74250084	0.85933705
OTZ 5	1.07771433	1.24588966	1.29654239
OTZ 10	1.4412242	1.73464458	1.17690758
OTZ 25	1.37294791	1.76358696	1.47348512

Dose response after WG treatment

	exp1	exp2	exp3	exp4
SFN	5.56525097	2.12301741	6.49317677	1.78536585
DMSO	1	1	1	1
WG 10	1.4327335	1.2410329	1.20663696	1.25001544
WG 25	1.71591096	1.46879607	1.96884058	1.04167953
WG 50	2.49262116	1.28384279	2.80483092	1.70948758
WG 75	3.59292303	2.03207354	5.83022774	2.74308943

Data for Fig S2: cell viability

	exp1	exp2	exp3	mean	SEM
DMSO	100	100	100	100	0
SFN	98.78571	92.78794	96.51526	96.0296367	1.74835006
WG	99.53571	90.63509	75.55876	88.57652	6.99766189
OTZ	98.35714	92.11518	98.05335	96.1752233	2.03191502
DMF	108.9643	109.1496	90.98774	103.03388	6.02330753
